# Some Issues of Shrinkage-Reducing Admixtures Application in Alkali-Activated Slag Systems

**DOI:** 10.3390/ma9060462

**Published:** 2016-06-10

**Authors:** Vlastimil Bílek, Lukáš Kalina, Radoslav Novotný, Jakub Tkacz, Ladislav Pařízek

**Affiliations:** Materials Research Centre, Faculty of Chemistry, Brno University of Technology, Brno 612 00, Czech Republic; kalina@fch.vut.cz (L.K.); xcnovotny2@fch.vut.cz (R.N.); tkacz@fch.vut.cz (J.T.); xcparizekl@fch.vut.cz (L.P.)

**Keywords:** alkali activated slag, shrinkage reducing admixture, shrinkage, hydration, microstructure, retardation

## Abstract

Significant drying shrinkage is one of the main limitations for the wider utilization of alkali-activated slag (AAS). Few previous works revealed that it is possible to reduce AAS drying shrinkage by the use of shrinkage-reducing admixtures (SRAs). However, these studies were mainly focused on SRA based on polypropylene glycol, while as it is shown in this paper, the behavior of SRA based on 2-methyl-2,4-pentanediol can be significantly different. While 0.25% and 0.50% had only a minor effect on the AAS properties, 1.0% of this SRA reduced the drying shrinkage of waterglass-activated slag mortar by more than 80%, but it greatly reduced early strengths simultaneously. This feature was further studied by isothermal calorimetry, mercury intrusion porosimetry (MIP) and scanning electron microscopy (SEM). Calorimetric experiments showed that 1% of SRA modified the second peak of the pre-induction period and delayed the maximum of the main hydration peak by several days, which corresponds well with observed strength development as well as with the MIP and SEM results. These observations proved the certain incompatibility of SRA with the studied AAS system, because the drying shrinkage reduction was induced by the strong retardation of hydration, resulting in a coarsening of the pore structure rather than the proper function of the SRA.

## 1. Introduction

Ordinary Portland clinker or cement (OPC)-based binders are probably the most common in concrete production. OPC is a traditional and versatile binder but, on the other hand, its manufacturing consumes great amounts of energy and significantly contributes to the global emissions of greenhouse gases. Approximately one ton of CO_2_ is released per one ton of cement produced [1]. Therefore, it is necessary to search for some alternative binders such as calcium aluminate cements, calcium sulfoaluminate cements or supersulfated cements [2]. Another possible way is the formulation of alkali-activated binders, usually based on blast furnace slag (BFS), fly ash (FA) or metakaolin [3]. According to Duxson *et al.* [4], geopolymers can provide approximately 80% reduction of CO_2_ emissions compared to OPC.

AAS-based materials seem to be promising for practical applications. Naturally, their properties are often compared to those of OPC-based materials. In general, AAS can be equal to or even better than OPC in terms of mechanical strength [5], durability in aggressive environments [6,7,8], behavior at elevated temperatures [9,10,11] and interfacial transition zone [12]. However, AAS-based materials also have some drawbacks, especially their complicated workability improvement by conventional superplasticizers designed for OPC [13,14], rapid setting [15] and high shrinkage [15,16].

Shrinkage of AAS can be classified into drying shrinkage, autogenous shrinkage and carbonation shrinkage [17]. The considerably higher shrinkage of AAS compared to that of OPC is mostly attributed to the significantly higher mesopore content in AAS binders [18] and the different nature of C–S–H or C–S–A–H gel [19]. Autogenous shrinkage can be regarded as the macroscopic result of the effects of chemical shrinkage and self-desiccation [20]. Lee *et al.* [21] stated that self-desiccation is the main reason for the autogenous shrinkage of AABFS/FA. Shrinkage during drying originates from the two mechanisms of capillary suction and disjoining pressure. The former dominates for pore diameters higher than 10 nm, while the latter becomes more important for a very fine porosity [22].

Recently, many various efforts to reduce the shrinkage of AAS were made, such as the partial replacement of BFS by mineral additives such as FA [21,23,24,25] and silica fume [23] or a combination of these [23], initial curing at elevated temperature [26,27,28,29], internal curing [30], use of fibers [31,32,33] and utilization of some expanding admixtures [34,35,36]. The most effective seem to be the partial replacement of slag by silica fume and heat curing prior to dry air exposure. However, heat curing means additional energy consumption and is restricted mostly to the precast production. Moreover, volume changes during the heat curing stage are not always measured, despite the fact that they can noticeably contribute to the total shrinkage, particularly under sealed conditions.

Another possibility of shrinkage mitigation is provided by SRAs. The use of these admixtures in order to reduce both autogenous and drying shrinkage of OPC-based matrices is relatively well established [37,38,39,40,41] and such admixtures are commercially available. The beneficial effect of SRAs is attributed mainly to the decline of the surface tension of the pore water. Only a few studies investigating the influence of these admixtures, designed originally for OPC systems, on properties of AAS were reported: Puertas and Palacios [42] reduced both autogenous and drying shrinkage of AAS by using SRA based on polypropylene glycol. Significantly higher shrinkage reduction was observed at 99% relative humidity than at 50% relative humidity. Better results for moist curing than for dry curing were reported by Bilim *et al.* [43], also using SRA based on polypropylene glycol. Bakharev *et al.* [44] significantly reduced shrinkage by using some nonstandard SRA and then reduced it even more by using an air-entraining admixture, which was recommended for use in AAS concrete, as it greatly improved workability.

Despite the great effort and promising results mentioned above, shrinkage is still regarded as the main limiting factor for the practical use of AAS. The aim of this paper is to investigate the effect of different types of SRAs from those mentioned above not only on the shrinkage of AAS, but also on its other properties such as microstructural and strength development.

## 2. Materials and Methods

### 2.1. Materials and Sample Preparation

BFS from the Czech production with a volume mean diameter of about 12 μm was used as a solid precursor for the reference binder.

The prevailing amount of amorphous phase was determined by X-ray diffraction as well as the presence of melilite, merwinite and traces of β-C_2_S and calcite. The chemical composition of slag determined by X-ray fluorescence is given in Table 1.

BFS was activated by waterglass with a silica modulus of 1.85 provided by Vodní sklo, a.s. The amount of waterglass was adjusted to maintain the mass ratio Na_2_O/slag of 0.04. Commercially available SRA based on 2-methyl-2,4-pentanediol originally designed for OPC systems was used to test its effect on the properties of AAS in amounts of 0.25, 0.50 and 1.0 wt. % of slag. Corresponding mortars and pastes were marked as SRA0.25, SRA0.50 and SRA1.0, respectively. SRA was mixed with activating solution just before the slag addition. Specimens with no SRA were also prepared as a reference (R). The mixture of SRA with activating solution quickly separated into two layers.

Based on the composition described above, both AAS pastes and mortars were prepared. In the case of the mortars, three different fractions of siliceous Czech standard sand (complying with ČSN EN 196-1) were used as a fine aggregate. The sand-to-binder ratio was 2:1. The water-to-binder ratio (w/b) was 0.35 for the pastes and 0.40 for the mortars. After the mixing, fresh material was cast into the steel molds and moist-cured for 24 h. After that, specimens were demolded and water- and/or air-cured until the time of testing (see following sections). Both mixing and curing was performed at laboratory temperature.

### 2.2. Drying Shrinkage Tests

Drying shrinkage tests were based on ASTM C596. After three days of water curing, mortar bars with dimensions of 25 mm × 25 mm × 285 mm were removed from water and air-cured at laboratory conditions, *i.e.*, relative humidity of approximately 50% and temperature of 23–25 °C, until the age of 28 days. During this period, relative length changes were measured almost every workday using the ASTM C490 apparatus. Three samples of each mixture were measured. For comparison, mortars with 0% and 1% of SRA were also water-cured until the age of 28 days and then the relative length changes during air-curing were measured.

### 2.3. Mechanical Strength Testing

Flexural strengths were tested on the mortar specimens with dimensions of 20 mm × 20 mm × 100 mm. Compressive strength tests were performed on the broken parts after the flexural strength tests. Only water-cured samples were used for the mechanical strength tests. These tests were performed at the age of 24 h, seven days and 28 days. Three prisms were used for every flexural strength test.

### 2.4. Isothermal Calorimetry

Influence of SRA on AAS hydration at 25 °C was investigated through isothermal conduction calorimetry. Slag and mixture of liquid components of the AAS paste were tempered separately inside the calorimeter, mixed together and mechanically stirred for three minutes. This *in situ* mixing enabled an immediate heat flow measurement.

### 2.5. Mercury Intrusion Porosimetry

MIP measurements were conducted on the paste samples containing 0% and 1% of SRA at the age of one, seven, 28 and 56 days. These pastes were moist-cured during the first 24 h and then cured immersed in water. Before the MIP testing, thin plates with dimensions of approximately 10 mm × 30 mm × 3 mm were sawed from inner parts of the 20 mm × 20 mm × 100 mm prisms and put into acetone in order to stop hydration until their drying and start of MIP measurement. Three such plates were used together for one measurement. Pore diameter in the range of 7 nm to 100 μm was calculated according to the Washburn equation [45] from the pressure required to intrude pores of a certain diameter. The contact angle between mercury and a pore wall was assumed to be 140° and the surface tension of mercury was assumed to be 480 mN/m.

### 2.6. Scanning Electron Microscopy

For the SEM observations in the secondary electron mode the same paste prisms with the same curing conditions as for the MIP measurements were prepared, but unlike the MIP measurements, SEM observations were performed on the fracture surfaces. An accelerating voltage of 10 kV was used. Broken parts of hardened pastes were immersed in acetone in order to stop hydration until the time of testing. Then the samples were stuck on a carbon tape and the exposed fracture surfaces were sputter-coated with gold. SEM tests were performed at the age of 24 h, three, 14 and 28 days.

## 3. Results and Discussion

### 3.1. Drying Shrinkage and Weight Loss

**Figure 1:**
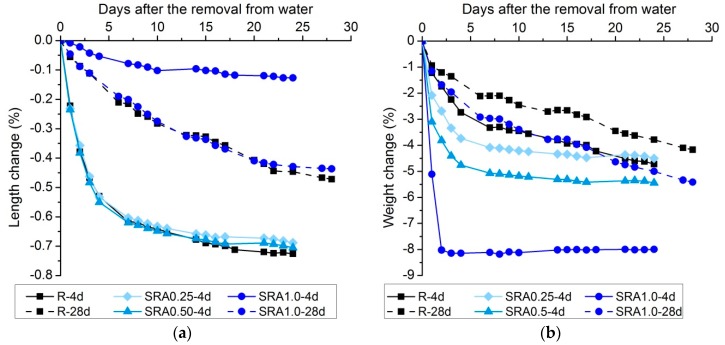
Effect of SRA on (**a**) drying shrinkage development and (**b**) weight loss development during drying of AAS mortars.

The drying shrinkage development depending on the mortar composition and the age of the samples at the end of water curing (four or 28 days) is presented in Figure 1a. It can be seen that SRA at the dosages of 0.25% and 0.50% did not markedly affect the drying shrinkage of the plain mortar (R), but when 1% of SRA was used, shrinkage was reduced by more than 80%. When more mature mortars were exposed to the atmospheric conditions, there was no difference between the drying shrinkage of the plain (R-28d) mortar and that containing 1% of SRA (SRA1.0-28d). This indicated that the beneficial effect of SRA on shrinkage diminished as the time of water curing increased. This may be related to the leaching of the SRA molecules during the water curing. The leaching phenomenon of SRA in OPC-based materials was widely studied by Eberhardt [46], where the vanishing of the beneficial effect of the SRA mobile fraction in the capillary range of humidity was observed. Also, the differences in microstructural development could be an issue, as will be discussed in Section 3.4 and Section 3.5. A decrease in shrinkage of the reference mortar with a prolonged time of curing is in agreement with data summarized in [3] and is probably attributed to the maturity of the binder phase.

Figure 1b shows the effect of SRA on weight changes during drying. The increasing amount of SRA resulted in an increase in weight loss, especially during the initial stages of drying. When the length of water curing was increased, a lower weight loss was recorded for both the plain and the SRA mortar. The reason is a decrease in porosity and more water incorporated in a binder phase as a result of the proceeding hydration reactions. However, unlike similar shrinkage of these mortars after prolonged curing, the weight loss values of S1.0-28d mortar were significantly higher than those of the R-28d mortar. Higher weight losses of mortars with SRA than those without were observed also for OPC-based materials and this was explained by the lower liquid saturation for the same relative humidity induced by the SRA [47]. Contrary to this, Saliba *et al.* [38] observed lower mass loss during the drying of concrete with SRA compared to the concrete without, at least during the first 24 h. Unfortunately, there is a lack of information about the effect of SRAs on the weight loss of AAS in the literature to compare the obtained results. Nevertheless, as the chemical admixtures are usually consumed during the proceeding hydration reactions, it is more likely that the difference after 28 days of water curing is a consequence of the different porosity of the binder phase. This is also in agreement with the decrease in strength and the increased volume of the intruding mercury for specimens with SRA, as will be shown further.

### 3.2. Flexural and Compressive Strength

**Figure 2:**
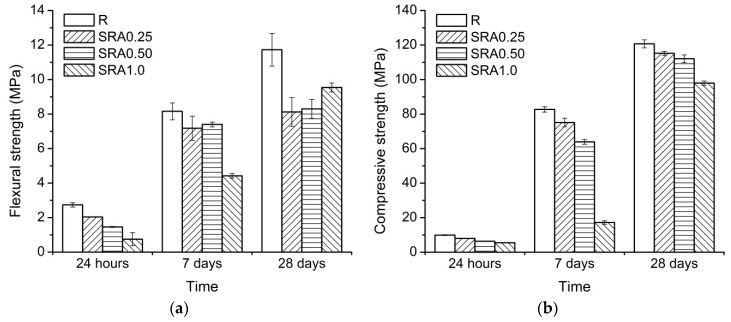
Effect of SRA on (**a**) flexural strength and (**b**) compressive strength development of AAS-based mortars; error bars correspond to a standard error of the mean.

The flexural strength development of the water-cured mortars is shown in Figure 2a. It is clear that even a low amount of SRA resulted in a significant decrease in the flexural strength. This effect increased with the increasing SRA dosage, especially at early ages. At the age of 28 days, all three SRA-containing mixtures showed similar flexural strengths whose values were about 30% lower than that of the reference mortar. For more precise results, more than three specimens of each series should be tested.

As can be seen from Figure 2b, compressive strength values showed a similar trend as to what was observed for flexural strength development, *i.e.*, with an increasing amount of SRA the compressive strength decreased. This was recorded for all the tested series. While compressive strength development for the mortars with 0.25% and 0.50% of SRA had a comparable shape to the reference mortar, the SRA1.0 mortar showed a different rate of strength gain. For up to seven days, the SRA1.0 mortar had a very low compressive strength, but beyond this age, it had the highest strength gain of all the tested mortars, which may indicate that SRA caused some retardation during the hydration processes. We observed a similar effect on the compressive strength development of AABFS/FA mortars for the three different commercially available SRAs based on modified alcohols [48]. In order to investigate this feature, additional experimental methods, namely isothermal calorimetry, MIP and SEM, were applied.

### 3.3. Isothermal Calorimetry

**Figure 3:**
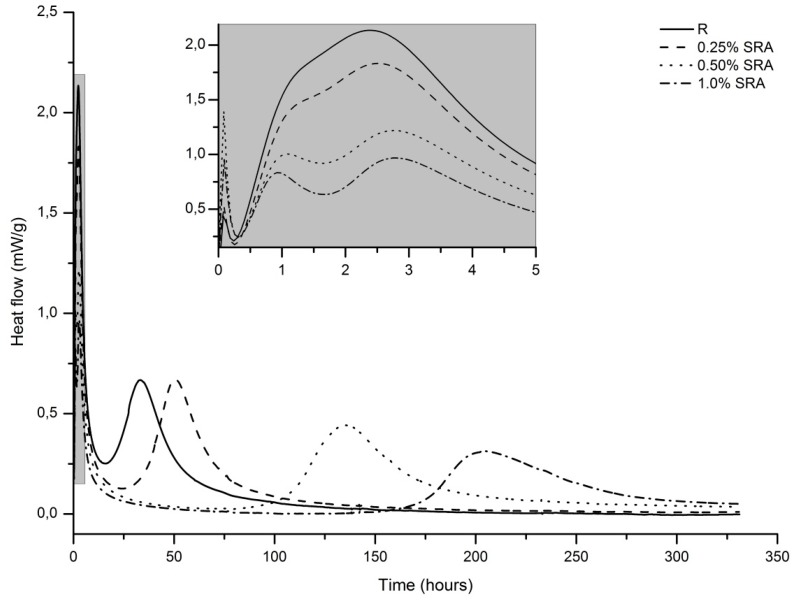
Effect of SRA on hydration heat evolution of AAS pastes.

The heat flow evolution curves for the plain and SRA-containing mortars are shown in Figure 3. Especially in the case of the plain slag paste curve, the typical shape for waterglass-activated slag was observed, as was schematically introduced by Shi and Day [49], *i.e.*, the initial peak was related to the particle wetting and slag dissolution as well as the additional initial peak was mainly associated with the primary C–S–H gel formation, both during the pre-induction period. Then several hours of the induction period followed, after which the acceleration/deceleration stage occurred, where massive precipitation of the reaction products took place. In the presence of SRA, similar curves with some differences were recorded.

Firstly, the initial peak seems to be more intense when the SRA is added, which might be caused by the interactions of the SRA molecules with the AAS system, e.g., the adsorption of SRA molecules onto the slag particles. However, the reproducibility of this peak is generally poor, which makes its interpretation uncertain. This is also probably the reason why the most intense peak was recorded for the paste with 0.5% of SRA.

Secondly, significant changes of the additional initial peak were observed. With the increasing SRA content, the overall intensity of this peak decreased, but what is even more interesting is that this peak was split into two partially mutually overlapping peaks with its maximums just before 1 and 3 h, respectively. For the plain slag paste curve, these peaks were merged into one peak, but even in this case two parts with different slopes of the increasing heat evolution rate are distinguishable. This could imply that the formation of the primary C–A–S–H gel proceeded in two steps, or some additional process occurred. Increasing the amount of SRA in the pastes made this observation more obvious and led even to the splitting of this peak into two partially separated peaks. To the best knowledge of the authors, this has not been reported yet and deserves further study.

Thirdly, the occurrence of the peak of the acceleration/deceleration stage is strongly affected by the dose of SRA. The higher the SRA portion, the more delayed this peak, which indicates a retardation effect of SRA on the AAS hydration. Moreover, the peak was wider and lower in magnitude when the dose of the SRA was increased, which could be due to the more developed surface layer of the reaction products on the slag grains and, consequently, the slower diffusion of pore solution species. A similar effect, although much less intense, was reported for slag systems activated with an increasing modulus of waterglass [50], *i.e.*, a decreasing alkalinity of the activating solution, but such a long induction period as in this case cannot be explained only by the very slight neutralization and therefore by the increase of the silicate modulus of the activating solution by the SRA (pH approx. 6.5). The reason for this could be some process occurring in the pre-induction period during or just after the mixing, because further calorimetric response is significantly affected, the formation of the primary C–A–S–H gel is suppressed and the formation of the secondary C–A–S–H gel is delayed. The exact retardation mechanism deserves further study. A certain retardation effect of SRA based on polypropylene glycol on the hydration of waterglass-activated slag was reported Palacios and Puertas [42], but unlike the SRA used in the present study, their SRA delayed the main hydration peak only by several hours. It also seems that increasing the dose of their SRA resulted in the more intense second peak in the pre-induction period, while in the present study SRA had the opposite effect. Therefore, two different SRAs may affect AAS hydration in a completely different manner.

**Figure 4:**
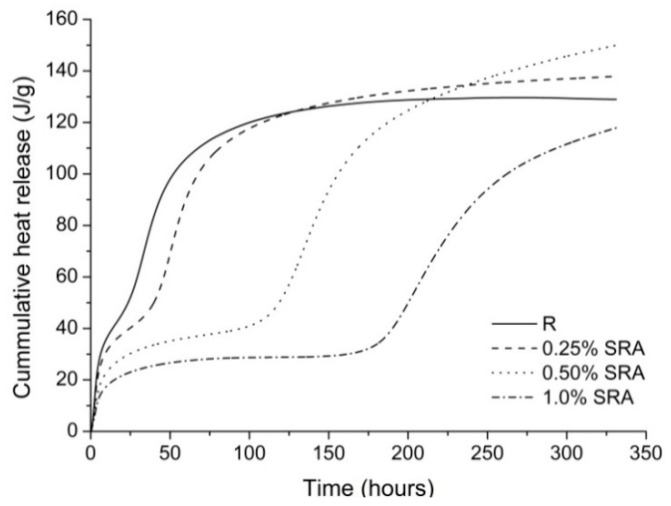
Effect of SRA on the total heat released during the hydration of AAS pastes.

The retardation effect of SRA on AAS hydration is also evident from Figure 4, which shows the cumulative heat release of the evaluated pastes. Although during the first days of hydration the highest amount of the heat released was recorded for the pure slag paste, the total heat release increased in the presence of the SRA. This indicates that after the retardation, hydration reactions can continue to an even greater extent than for the pure slag paste. Nevertheless, the strengths measured up to 28 days of hydration were still significantly lower when SRA was added to the slag paste.

The action of chemical admixtures can be negatively affected by the high pH in AAS, as was proved by Palacios and Puertas [13], particularly for superplasticizers. On the other hand, this was not the case for the SRA in their study. Also, the SRA used in this study would be stable in a highly alkaline environment, but the deprotonation of its hydroxyl groups as a consequence of high pH can be expected, despite the glycols belonging to the non-ionic surfactants [47]. Therefore, one cannot exclude the adverse effect of SRA on AAS properties resulting from either possible adsorption on solid particles or interactions with the dissolved species present in AAS pore solution.

### 3.4. Mercury Intrusion Porosimetry

Since there are serious limiting factors in cementitious systems for the use of the Washburn equation adopted for the pore size distribution calculations, particularly the inaccessibility of all the pores for mercury, MIP cannot provide realistic pore size distribution determination [51]. However, it can give us interesting information about the size of the pores (threshold diameter), which interconnect larger pores throughout the matrix. The threshold pore diameter is related to the permeability and diffusion characteristics of the material and thus may affect its durability [52].

**Figure 5:**
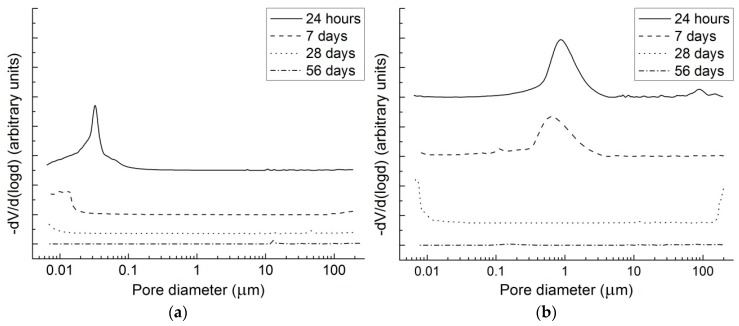
MIP curves showing the effect of SRA on pore structure development: (**a**) pure AAS paste; (**b**) AAS paste with 1% of SRA.

Differential intrusion curves obtained for the reference paste and the paste containing 1% of SRA after the different hydration times are shown in Figure 5. A great difference between the pore structures of these two mortars can be observed, particularly at the early ages: the threshold diameter of the reference paste after 24 h (approx. 70 nm) was much lower than that of the SRA paste (more than 1 μm). Similarly, after seven days the SRA paste still exhibited the net of relatively large pores, while significant mercury intrusion into the pure slag paste was recorded only for pores corresponding to a pore diameter lower than 20 nm. At later ages, the reference and SRA pastes’ intrusion curves resemble each other more. This could be a consequence of the proceeding of retarded hydration reactions as was shown in the previous section and also of the gradual leaching of SRA, as the specimens were cured in water. For both pastes no intrusion was observed after the 56 days, indicating pore system refinement beyond the measurability range of MIP. Also pore blockage, as a consequence of the hydration products’ growth, may affect the volume of the intruded mercury.

Such high differences between the intrusion curves of the studied pastes during the first several days of hydration proved that the presence of 1% SRA caused the retardation of the hydration products’ formation and thus slowed down the pore structure refinement. Therefore, if these pastes or mortars are exposed to dry ambient conditions without sufficient length of the curing stage, they start to dry rapidly and are not able to fully hydrate. This is the main reason for such an intense weight loss of SRA1.0 mortar during drying as presented in Section 3.1. The coarser pore structure of SRA1.0 specimens compared to those without SRA led to a modified weight loss development, with accelerated weight loss rate during the first days of drying. A slight increase in weight beyond the third day of dry air exposure is probably caused by the intense carbonation which was, together with the relatively low strengths of these air-stored specimens, presented in [53]. The importance of the pore size distribution for drying shrinkage was emphasized by Collins and Sanjayan [18] and it can thus be concluded that the great reduction of drying shrinkage induced by 1% of SRA is attributed to coarser pores, where the shrinkage forces are not so high. Puertas and Palacios [42] also explained the beneficial effect of SRA based on polypropylene glycol on shrinkage with the pore structure redistribution, but as they stated, it was “due to the decrease of the capillary stress of the water that SRA induces during the mixing process”. This is the difference between their study and the present one, where the SRA induced changes in the pore structure primarily by the retardation of hydration.

### 3.5. Scanning Electron Microscopy

**Figure 6:**
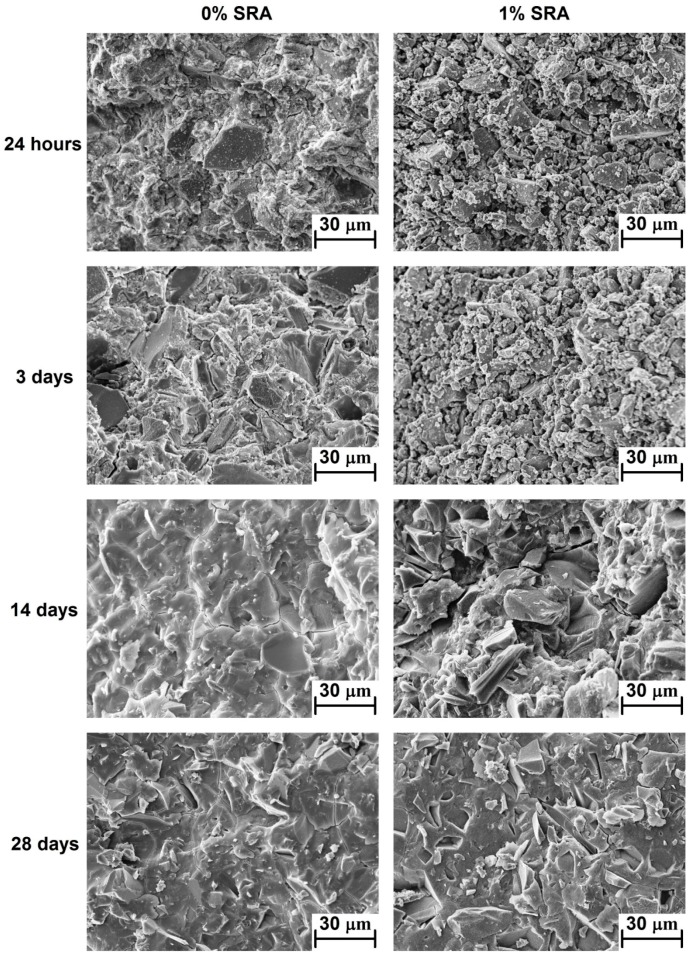
SEM image showing the effect of SRA on the microstructure development of AAS paste.

The data obtained from the previously described methods are in a good agreement with the results from SEM presented in Figure 6. Microstructural development of the pastes with 0% and 1% of SRA was investigated. After the 24 h of hydration, the reference mortar showed a relatively dense matrix surrounding the slag grains, while in the case of SRA-containing paste only surface reacted separate slag grains were observed. After three days, the situation was very similar to that after 24 h, *i.e.*, dense C–A–S–H gel formed in the plain paste in contrast with the porous structure and practically unreacted slag grains. The situation of the SRA paste at the ages of 14 and 28 days was completely different, where its microstructure was the same as that of the paste with no SRA addition. This agrees well with the experimental data either from the strength testing or calorimetry or MIP measurements.

## 4. Conclusions

This study investigated the effect of SRA based on 2-methy-2,4-pentanediol on the mechanical properties, drying shrinkage and microstructure development of waterglass-activated slag (M_S_ = 1.85, Na_2_O/slag = 0.04).

It was found that the SRA greatly influenced the course of hydration of the studied AAS system. An increase of the SRA content resulted in a reduction and bisection of the second peak on the calorimetric curve and also in a delayed occurrence of the main hydration peak.

This was observed as flexural and compressive strength reduction, increased weight loss during drying and coarser porosity of mixtures with the SRA when compared to the mixtures without the SRA, particularly during the first several days of hydration. This is also probably the main reason for such a high shrinkage reduction recorded for mortar with 1% of SRA exposed to the dry environment four days after mixing, *i.e.*, before reaching the proper degree of hydration.

## Figures and Tables

**Table 1:** Chemical composition of BFS as determined by XRF.

Raw Material	Chemical Composition wt. %									
BFS	SiO_2_	Al_2_O_3_	CaO	Na_2_O	K_2_O	MgO	SO_3_	Fe_2_O_3_	TiO_2_	MnO
	34.7	9.1	41.1	0.4	0.9	10.5	1.4	0.3	1.0	0.6

## Abbreviations

The following abbreviations are used in this manuscript:

AAS	Alkali activated slag
AABFS/FA	alkali activated blend of BFS and FA
BFS	Blast furnace slag
C–S–H	Calcium–silicate–hydrate
C–A–S–H	Calcium–aluminate–silicate–hydrate
FA	Fly ash
MIP	Mercury intrusion porosimetry
OPC	Ordinary Portland cement
SRA	Shrinkage reducing admixture
SEM	Scanning electron microscopy
